# Delayed-Onset Autoimmune Hemolytic Anemia in Advanced HIV With Cerebral Toxoplasmosis

**DOI:** 10.7759/cureus.106704

**Published:** 2026-04-09

**Authors:** Mohammed F Alahmadi, Oweida Aldosary, Amer Bugnah, Hamad Alyami

**Affiliations:** 1 Department of Internal Medicine, King Saud Medical City, Riyadh, SAU; 2 Department of Infectious Diseases, King Saud Medical City, Riyadh, SAU; 3 Department of Hematology and Oncology, King Saud Medical City, Riyadh, SAU

**Keywords:** advanced hiv disease, autoimmune hemolysis, autoimmune hemolytic anemia (aiha), cerebral toxoplasmosis, hiv aids

## Abstract

Autoimmune hemolytic anemia (AIHA) is an uncommon immune-mediated complication of human immunodeficiency virus (HIV) infection and represents a rare cause of anemia in this population. While anemia in HIV is typically multifactorial, AIHA has most often been reported early in the disease course or around the initiation of antiretroviral therapy. Delayed-onset AIHA developing during prolonged hospitalization is rarely described, particularly in patients with advanced immunosuppression.

We report a case of delayed-onset warm AIHA occurring more than two months after admission in a patient with newly diagnosed advanced HIV infection complicated by cerebral toxoplasmosis. The patient developed acute hemolysis with laboratory evidence of immune-mediated destruction of erythrocytes after a period of clinical stability. Secondary causes of hemolysis were excluded, and the patient demonstrated a favorable response to corticosteroid therapy without relapse.

This case highlights that AIHA may occur late during hospitalization, including during periods of partial immune recovery following antiretroviral therapy. Clinicians should maintain a high index of suspicion for immune-mediated hemolysis in patients with HIV who develop unexplained anemia. Further studies are needed to better understand the mechanisms underlying delayed-onset AIHA and its relationship to immune reconstitution.

## Introduction

Autoimmune hemolytic anemia (AIHA) is an uncommon but clinically significant immune-mediated complication of human immunodeficiency virus (HIV) infection. Anemia is a frequent hematologic abnormality in patients with HIV, and in most cases, it is multifactorial, resulting from opportunistic infections, nutritional deficiencies, chronic inflammation, bone marrow suppression, or medication effects [1,2].

AIHA represents a rare cause of anemia in this population and accounts for only a small proportion of hematologic complications in patients with HIV infection [3-5]. The pathogenesis of AIHA in HIV is incompletely understood but is thought to involve immune dysregulation associated with chronic viral infection. Proposed mechanisms include polyclonal B-cell activation, molecular mimicry between viral and erythrocyte antigens, and immune reconstitution following initiation of antiretroviral therapy, all of which may promote the production of autoantibodies against red blood cells [6].

Most reported cases of HIV-associated AIHA occur early in the disease course or around the time of HIV diagnosis or antiretroviral therapy initiation [3,7,8]. Delayed-onset AIHA developing late during prolonged hospitalization for opportunistic central nervous system infection is rarely described, particularly in patients with profound CD4 depletion. We report a case of delayed-onset warm AIHA occurring more than two months into hospitalization in a patient with newly diagnosed advanced HIV infection and severe immunosuppression.

## Case presentation

**Figure 1:**
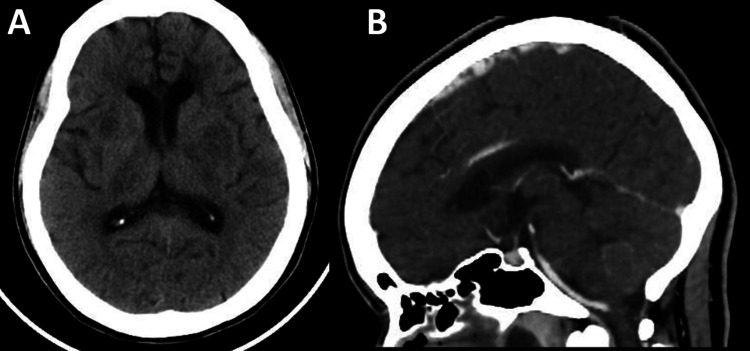
(A) Noncontrast brain computed tomography demonstrating no acute intracranial hemorrhage or mass effect. (B) Computed tomography cerebral venography demonstrating patent dural venous sinuses without evidence of venous sinus thrombosis

A woman in her forties with no known past medical history was found unconscious and brought to the emergency department. No family members or acquaintances were available to provide collateral history. On presentation, she was febrile, stuporous, and aphasic, with right-sided weakness. Initial evaluation, including brain computed tomography (Figure 1A), cerebral venography (Figure 1B), and lumbar puncture, suggested meningoencephalitis, and empiric antimicrobial therapy was initiated.

**Figure 2:**
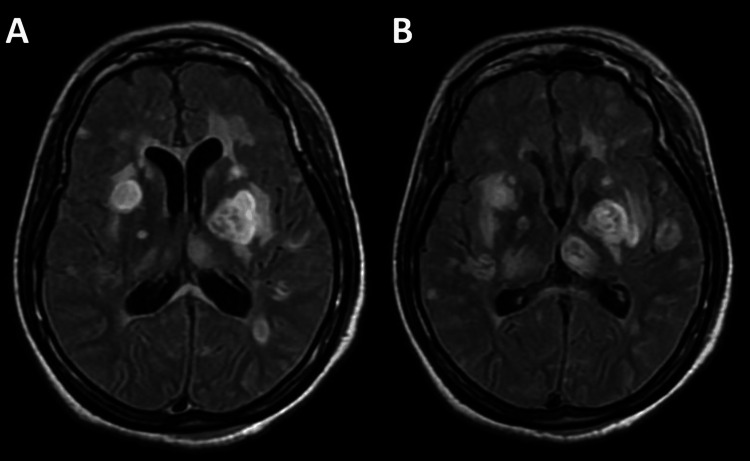
Brain MRI, axial T2/FLAIR images MRI: magnetic resonance imaging; FLAIR: fluid-attenuated inversion recovery; HIV: human immunodeficiency virus (A) Hyperintense lesion involving the basal ganglia with surrounding vasogenic edema. (B) Additional multifocal hyperintense lesions in the deep white matter, consistent with cerebral toxoplasmosis in the setting of advanced HIV infection

Further investigations revealed a newly diagnosed HIV type 1 infection with a viral load of 5,130,000 copies/mL and a CD4 count of 7 cells/µL. Brain magnetic resonance imaging demonstrated multiple ring-enhancing lesions (Figures 2A-2B), and positive Toxoplasma immunoglobulin G serology supported a diagnosis of cerebral toxoplasmosis. The patient developed severe neurological and cognitive impairment, requiring prolonged hospitalization, and remained bedbound throughout her hospital stay. She was treated with trimethoprim-sulfamethoxazole for cerebral toxoplasmosis and *Pneumocystis jirovecii* pneumonia, followed by transition to a prophylactic dose. Antiretroviral therapy was initiated on day 18 of hospitalization.

Her hemoglobin remained stable between 8.5 and 9 g/dL during the first eight weeks of hospitalization. On day 62, repeat testing showed a marked decline in HIV viral load to 1,530 copies/mL (previously 5,130,000 copies/mL) and an increase in CD4 count to 39 cells/µL. On day 65 of admission, she developed fever (highest 39.3°C), scleral icterus, and tea-colored urine, accompanied by a rapid decline in hemoglobin to 6.2 g/dL. Laboratory findings at the time of hemolysis are summarized in Table 1, while additional investigations to exclude other causes of hemolytic anemia and assess immune status are presented in Table 2.

**Table 1:** Laboratory findings at the time of hemolysis

Test	Result	Reference range
Hemoglobin (day 64 of hospitalization)	8.3 g/dL	12-16 g/dL
Hemoglobin (day 65 of hospitalization)	6.2 g/dL	12-16 g/dL
Reticulocyte count	7.80%	0.5-2.5%
Lactate dehydrogenase	2624 U/L	125-220 U/L
Indirect bilirubin	17 µmol/L	<12 µmol/L
Haptoglobin	<8 mg/dL	30-200 mg/dL
Direct antiglobulin test	Positive	Negative
Schistocytes (peripheral smear)	7-8%	Absent
Platelet count	218 × 10^3^/µL	150-400 × 10^3^/µL
Creatinine	75.3 µmol/L	50.4-90.1 µmol/L
Urinalysis (red blood cells)	5-10 per high-power field	0-2 per high-power field

**Table 2:** Additional investigations ANA: antinuclear antibody; anti-dsDNA: anti-double-stranded DNA antibody; anti-Smith: anti-Smith antibody; anti-SSA: anti–Sjögren’s syndrome-related antigen A (Ro) antibody; anti-SSB: anti–Sjögren’s syndrome-related antigen B (La) antibody; anti-RNP: anti–ribonucleoprotein antibody; anti-Scl-70: anti-topoisomerase I (Scl-70) antibody; anti-Jo-1: anti–histidyl-tRNA synthetase (Jo-1) antibody

Test	Result	Reference range
Glucose-6-phosphate dehydrogenase activity	12.52 U/gHb	5.0-16.4 U/gHb
HIV viral load (day 62 of hospitalization)	1,530 copies/mL	-
CD4 count (day 62 of hospitalization)	39 cells/µL	400-1300 cells/µL
Cytomegalovirus PCR	362 copies/mL	34.5-10,000,000 copies/mL
Blood cultures (two sets)	Negative	Negative
Sputum culture	Negative	Negative
Urine culture	Negative	Negative
Malaria smear	Negative	Negative
Hepatitis B antigens	Negative	Negative
Hepatitis C antibody	Negative	Negative
Autoimmune panel (ANA, anti-dsDNA, anti-Smith, anti-SSA, anti-SSB, anti-RNP, anti-Scl-70, anti-Jo-1)	Negative	Negative
Complement C3	0.97 g/L	0.75-1.81 g/L
Complement C4	0.31 g/L	0.11-0.41 g/L

No new medications had been introduced prior to the onset of hemolysis. The patient was managed with supportive red blood cell transfusions and intravenous methylprednisolone at a dose of 1.5 mg/kg/day. Hemoglobin levels stabilized within 10 days of initiating corticosteroid therapy. Treatment was subsequently transitioned to oral prednisone at 1 mg/kg/day for two weeks, followed by gradual tapering. No additional immunomodulatory therapies were required.

No relapse of hemolysis was observed during the remainder of hospitalization. After 92 days of hospitalization, the patient was discharged to a long-term care facility due to persistent neurological and cognitive impairment. At the time of discharge, hemoglobin levels remained stable between 7.5 and 8 g/dL, without further transfusion requirements.

## Discussion

AIHA is an uncommon but recognized immune-mediated complication of HIV infection. While anemia in HIV is typically multifactorial, AIHA represents antibody-mediated destruction of erythrocytes and occurs in a small subset of patients [1-5]. The association between HIV infection and autoimmune cytopenias has been attributed to immune dysregulation, including polyclonal B-cell activation and loss of immune tolerance [6]. In advanced HIV infection, profound CD4 depletion may further contribute to impaired immune regulation and predispose to the development of autoantibodies.

In the present case, the patient had severe immunosuppression at presentation with a CD4 count of 7 cells/µL and a markedly elevated HIV viral load. Antiretroviral therapy was initiated during hospitalization, followed by a marked decline in viral load and partial CD4 recovery. The temporal relationship with antiretroviral therapy initiation, together with the marked decline in HIV viral load and partial CD4 recovery, raises the possibility that immune reconstitution inflammatory syndrome may have contributed to the development of AIHA. Such delayed immune shifts have been proposed as a mechanism for autoimmune complications in HIV infection, although the precise pathophysiology remains incompletely understood [6].

An additional diagnostic consideration in this case was thrombotic microangiopathy, particularly because schistocytes were observed on the peripheral blood smear. However, several features argued against this diagnosis. Despite the presence of approximately 7-8% schistocytes, the patient had preserved platelet counts and normal renal function, and there were no clinical features suggestive of microvascular thrombosis. In contrast, the presence of reticulocytosis, markedly elevated lactate dehydrogenase, undetectable haptoglobin, and a positive direct antiglobulin test strongly supported immune-mediated hemolysis. Infectious causes of hemolysis were also considered; although low-level cytomegalovirus viremia was detected, there was no clinical evidence of active cytomegalovirus disease to suggest a causative role in the hemolysis. Taken together, these findings favored a diagnosis of warm AIHA rather than microangiopathic hemolysis.

Previously reported cases of HIV-associated AIHA most commonly occur early in the disease course or around the time of HIV diagnosis or initiation of antiretroviral therapy [3,7,8]. In contrast, the present case is notable for the delayed onset of hemolysis occurring more than eight weeks after hospitalization, in the setting of partial immune recovery following antiretroviral therapy, with a significant decline in viral load and improvement in CD4 count. In addition, several previously reported cases have required second-line immunomodulatory therapies such as intravenous immunoglobulin or rituximab [6,8]. In comparison, our patient demonstrated a favourable and sustained response to corticosteroid therapy alone.

Current management guidelines recommend corticosteroids as first-line therapy for warm AIHA, with escalation to additional immunomodulatory agents in refractory cases [9]. Despite concerns regarding immunosuppressive therapy in patients with advanced HIV infection, our case illustrates that corticosteroid treatment can be effective and well-tolerated when secondary causes of hemolysis are carefully excluded.

Overall, this case highlights an unusual presentation of delayed-onset AIHA in the setting of advanced HIV infection, profound CD4 depletion, and prolonged hospitalization for opportunistic central nervous system infection. Recognition of immune-mediated hemolysis in this context is important because prompt diagnosis and appropriate therapy can lead to favourable hematologic outcomes even in severely immunocompromised patients.

## Conclusions

AIHA is a rare but clinically important complication of advanced HIV infection. This case demonstrates that AIHA may develop late during prolonged hospitalization, including during periods of partial immune recovery following antiretroviral therapy. Careful exclusion of secondary causes of hemolysis is essential, and timely initiation of corticosteroid therapy can result in favorable hematologic outcomes. Clinicians should maintain a high index of suspicion for immune-mediated hemolysis in patients with HIV who develop unexplained anemia during hospitalization.
